# Selenium-Binding Protein 1 in Human Health and Disease

**DOI:** 10.3390/ijms19113437

**Published:** 2018-11-02

**Authors:** Mostafa Elhodaky, Alan M. Diamond

**Affiliations:** Department of Pathology, University of Illinois at Chicago, Chicago, IL 60612, USA; melhod2@uic.edu

**Keywords:** selenium-binding protein 1, SBP1, SELENBP1, hSP56, cancer, disease

## Abstract

Selenium-binding protein 1 (SBP1) is a highly conserved protein that covalently binds selenium. SBP1 may play important roles in several fundamental physiological functions, including protein degradation, intra-Golgi transport, cell differentiation, cellular motility, redox modulation, and the metabolism of sulfur-containing molecules. SBP1 expression is often reduced in many cancer types compared to the corresponding normal tissues and low levels of SBP1 are frequently associated with poor clinical outcome. In this review, the transcriptional regulation of *SBP1*, the different physiological roles reported for SBP1, as well as the implications of SBP1 function in cancer and other diseases are presented.

## 1. Introduction

Selenium (Se) is a non-metallic, essential trace element for many organisms, including humans. Se has long been recognized for its potential in cancer prevention as evidenced by multiple animal, and human epidemiological studies that have reported an inverse association between Se status and cancer risk [1,2,3,4,5,6,7,8]. Many mechanisms have been suggested for the chemopreventive effect of Se [9,10,11], including DNA hypomethylation [12], blocked cell cycle progression, enhanced cell death, decreased cell proliferation, increased glutathione peroxidase or thioredoxin reductases activities [13], modulated ER stress response [14], and enhanced DNA repair [15]. Furthermore, Se has been found to play a key role in mammalian development [16] and immune function [17,18]. Low levels of Se may be a contributing factor to several pathologies, including male infertility [19], heart disease [20], inflammation [21,22], and neuromuscular disorders [23]. It is generally recognized that important cellular and organismal functions of Se are likely mediated by the action of selenoproteins constituting the mammalian selenoproteome [24]. While the functions of many selenoproteins are still unknown, they likely have a significant role in human health and disease. Human selenoproteins are generally classified into three categories [11,25]. The first category includes proteins in which Se is cotranslationally incorporated into the elongating peptide as the amino acid selenocysteine in response to an in-frame UGA codon in the corresponding messenger RNA [26]. The human selenoproteome contains 25 genes [25]. The second category consists of proteins in which Se is incorrectly substituted for sulfur in sulfur-containing amino acids due to the similarity in structure between these two elements. The third category is composed of selenium-binding proteins which bind Se by an unknown mechanism. This review will be primarily focused on one member of the latter category, selenium-binding protein 1 (SBP1, SELENBP1, hSP56).

## 2. SBP1 Discovery

SBP1 was first discovered in mouse liver in 1989 by Bansal et al. using ^75^Se labelling. Normal 6-week old female BALB/c mice were given a single intraperitoneal injection of ^75^Se in the form of Na_2_SeO_3_. After 40 h, animals were euthanized and livers were harvested for preparation of liver cytosols which were then used for a combination of gel filtration, ion-exchange chromatography and SDS-PAGE techniques. This led to identification of four selenium-binding proteins of apparent molecular weights of 12, 14, 24, and 56 kDa [27]. The 56-kDa protein was designated as SBP1, whereas the 24-kDa protein was identified as glutathione peroxidase 1 (GPX1), an enzyme that detoxifies hydroperoxides using reducing equivalents from glutathione [28]. The full-length human *SBP1* cDNA clone was first described by Chang et al. in 1997 and determined to be 1668 base pair (bp) long with an open reading frame encoding 472 amino acids [29]. *SBP1* is abundantly expressed in various human tissues, including liver, lung, prostate, colon, and pancreas, while moderate levels were detected in spleen, heart, and ovary. In contrast, its expression was barely detectable in thymus, testis, and peripheral blood leukocytes [30]. SBP1 is a highly-conserved protein. Flemetakis et al. reported that the predicted amino acid sequence of SBP1 is conserved in both plants and animals, ranging from 77 to 88% in plants, while the identity between the plants and mammalian proteins ranged from 57 to 60% [31]. By comparison, this degree of homology is higher than other conserved proteins, such as HSP60, γ-tubulin, apoptotic cell death 1 protein, and elF4E whose identities of the plant and human proteins are 44, 49, 48, and 52%, respectively [31]. The homology between the mammalian *SBP1* of mice and humans is 86% [31], indicating that the potential fundamental cellular and molecular functions for SBP1 are also conserved across different species. SBP1 is very similar to another selenium-associated protein, selenium liver binding protein (AP-56, SBP2), whose sequence differs by only 14 residues from SBP1 and is encoded by a distinct gene [32]. AP-56 is implicated in the detoxification of acetaminophen in the liver [32]. Although these genes are regulated differently, their similarity may indicate a role for SBP1 in detoxification.

## 3. The Role of Se in SBP1

The form of Se in SBP1 is currently unknown. Se is stably associated with SBP1, probably through a selenosulfide bond (perselenide), as indicated by the binding of Se to SBP1 being reversed by the addition of a reducing agent during SDS-PAGE [33]. Based on structural and functional studies, it was suggested that one cysteine in SBP1 was the likely binding site for the Se molecule, the cysteine at position 57 [34]. Converting cysteine 57 in SBP1 to a glycine and ectopically expressing that protein in human HCT116 cells that do not express detectable SBP1 levels indicated that the loss of the cysteine reduced the half-life of the protein, induced mitochondrial damage, and attenuated the degree of phosphorylation of signaling proteins such as p53 and GSK3β compared to the native protein expressed at similar levels [35].

The Se in SBP1 may facilitate its interaction with other proteins. SBP1 physically interacts with von Hippel–Lindau protein–interacting deubiquitinating enzyme 1 (VDU1), which plays a role in proteasomal protein degradation [33,36]. This indicates that SBP1, via its interaction with VDU1, may have a role in ubiquitination/deubiquitination-mediated protein degradation and detoxification pathways. When the Se moiety was dissociated from SBP1 by the addition of ß-mercaptoethanol, the interaction with VDU1 was completely blocked, indicating that Se may be essential for the interaction of these two proteins [33]. While the Se moiety is likely required for its interaction with VDU1, the inclusion of Se in SBP1 does not appear to be essential for functioning as methanethiol oxidase (MTO), a recently-discovered novel human SBP1 enzyme activity that metabolizes sulfur-containing molecules [37].

As a non-selenocysteine containing protein, SBP1 is not considered as a part of the “selenium hierarchy” that describes the relative response of selenoproteins to the availability of Se [38]. Initial studies feeding rats varying amounts of Se led to the conclusion that SBP1 levels were not likely dependent upon dietary Se supplementation [39]. However, there may be indirect regulation of SBP1 by Se due to its interaction with GPX1, a member of the selenocysteine-containing selenoproteins. GPX1 is a highly conserved and ubiquitously expressed enzyme that detoxifies hydrogen and lipid peroxides and is implicated in several diseases by human genetics [40]. There is a reciprocal regulatory relationship between SBP1 and GPX1. Ectopically expressing SBP1 in HCT116 human colon cancer cells that do not express endogenous SBP1 resulted in the inhibition of GPX1 enzyme activity without affecting protein levels [28], indicating a likely physical interaction. Consistent with this possibility was data indicating that knocking down *SBP1* in human liver cells resulted in a 4–5 fold increase in GPX activity, also without altering protein levels [41].

Expressing GPX1 in MCF7 human breast cancer cells that do not exhibit detectable GPX1 levels resulted in a decline in both SBP1 mRNA and protein levels [28]. The reciprocal relationship between SBP1 and GPX1 has also been established in mouse colon and duodenum epithelial cells [28], as well as human prostate and liver tissues [41,42]. This raises the possibility that SBP1 can be indirectly downregulated by Se because GPX1 is high on the Se hierarchy, being among the selenoproteins most responsive to Se availability. Support for the indirect regulation of SBP1 by GPX1 comes from experiments showing that increasing Se in the culture media of MCF7 cells caused a dramatic reduction in SBP1 levels only when GPX1 was present and GPX1 levels were increased by the Se supplementation [28].

## 4. SBP1 Levels Are Reduced in Cancer and Low Levels Are Predictive of Clinical Outcome

One of the striking observations about SBP1 is the diversity of the types of cancers in which SBP1 was found to be reduced compared to normal or benign tissues (reviewed in [43]), including cancers of the thyroid [44], lung [45], stomach [46,47], liver [41], kidney [48], ovary [49,50,51], breast [52], prostate [53,54], colon [55,56], head and neck [57], and malignant melanoma [58]. In addition to being lower in cancers, the degree of reduction of SBP1 in resected tissues is often predictive of how long a patient will be cancer free and survive their disease [43]. Reduced SBP1 levels have been correlated with poor survival in several types of carcinomas, including colorectal [55,59], gastric [47], nasopharyngeal [57], pulmonary [45], renal [48], and prostate [53] cancers. Recently, a search for genetic variations in selenoprotein genes revealed that a polymorphism in the gene for SBP1, along with variations in the genes of selenocysteine encoding genes, were associated with prostate cancer aggressiveness at diagnosis [60]. The exception to this pattern is ovarian cancer where higher levels of SBP1 were associated with poor survival [50].

**Figure 1:**
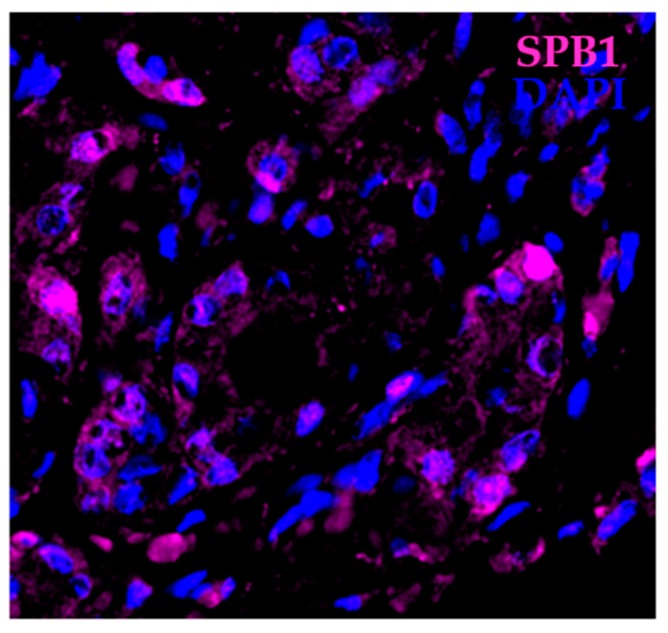
Localization of SBP1 in prostate cancer. Human prostate cancer tissue showing cells that express SBP1 (magenta) mostly in the cytoplasm and several cells that express SBP1 in the nucleus. Nuclei are highlighted with DAPI (blue).

In addition to its levels, the distribution of SBP1 between cellular compartments may be relevant to cancer etiology. The associations between prostatic SBP1 levels, tumor grade, and disease recurrence following prostatectomy were investigated using a tissue microarray containing tissue from more than 200 prostate cancer patients who experienced biochemical (PSA) recurrence after prostatectomy and matched control patients whose cancer did not recur [53]. Reduced SBP1 levels were associated with a higher likelihood of prostate cancer recurrence, as has been seen in other cancer types. The subcellular localization of SBP1 was both nuclear and cytoplasmic, with nuclear staining being sporadic (Figure 1). However, a lower nuclear-to-cytoplasmic distribution of SBP1 was associated with a higher tumor grade (Gleason score) [53]. These results indicate that sequestration of SBP1 in a particular cellular compartment may restrict access to relevant substrates or the protein has different functions at these locations.

### 4.1. Is SBP1 a Tumor Suppressor?

The frequent loss of SBP1 in cancer and the association of reduced SBP1 levels with greater mortality could imply that SBP1 is a tumor suppressor. Alternatively, its loss or downregulation may be a consequence of cancer development and progression, and the reduced levels represent a mere “bystander effect”. Data supporting the direct role of SBP1 in cancer comes from studies where its levels are altered in cells and consequences relevant to transformation and tumorigenesis are revealed. Over-expressing SBP1 in colon, gastric, and prostate cancer cells have generally yielded results consistent with a tumor suppressor function, including reduced growth in semi-solid media and decreased tumorigenicity in xenograft studies using immune-deficient mice [46,53,54,61,62]. When over-expressed in lung cancer cells, SBP1 reduced proliferation and induced greater apoptosis compared to control cells only when the cells were challenged with H_2_O_2_ [41]. Some of the phenotypic consequences of over-expressing SBP1 may be due to the downstream activation of the p53 tumor suppressor protein. Over-expression of SBP1 in HCT116 human colon cancer cells resulted in the increased phosphorylation of p53 [53]. In addition to the phosphorylation of p53, SBP1 over-expression in the same cells resulted in the differential expression of 132 proteins, many are associated with energy metabolism and MAPK, Wnt, NF-κB, and Notch signaling [61]. This same study reported that the expression of SBP1 resulted in the reduction of TWIST1, a critical regulator of the epithelial-mesenchymal transition and metastasis.

Consistent with over-expression data, either knocking down *SBP1* or inactivating the gene using CRISPR/Cas9 editing in mouse lung cancer cells and injecting these cells into syngeneic hosts increased the size of tumors obtained compared to controls, although the number of tumors was not increased [63]. Knockout mice that are null for SBP1 exhibit very limited pathology and do not develop tumors [64]. However, examining the ovaries from these animals by gene expression microarrays indicated the increased expression of several genes associated with ovarian carcinogenesis, including *Notch1* and *Fas1* [64]. Less clear is why tumor suppressor genes such as *Apc*, *RB1*, and *Trp53* were also enhanced in the ovaries from these mice. Collectively, studies altering the levels of SBP1 provide substantial evidence that SBP1 serves as a tumor suppressor and its loss or downregulation during cancer development contributes to disease development or progression.

### 4.2. Is SBP1 Downregulation an Early or Late Event in the Process of Tumorigenesis?

Given the data presented above indicating the frequent downregulation of SBP1 in cancers and its association with poor outcomes, it raises the issue of whether SBP1 loss occurs early in cancer development or late in the process, contributing to cancer progression. This issue was investigated by Zhang et al. who examined SBP1 levels in tissues classified as gastric cancer, precursor lesions, and matched controls of corresponding non-neoplastic epithelial tissues [65]. SBP1 was reduced in most of the gastric cancer tissues compared to its abundant expression in matched non-neoplastic controls and precursor lesions, including tissues obtained from gastric ulcers, gastric polyps, as well as tissues presenting with chronic atrophic gastritis, intestinal metaplasia and dysplasia [65]. SBP1 expression was similar in tissues with different levels of intestinal metaplasia or dysplasia indicating that the reduction of SBP1 levels may be a late event associated with gastric carcinoma progression from normal gastric epithelium or premalignant lesions [65]. These results are consistent with those of Kim et al. who observed much lower levels of SBP1 in colorectal carcinomas compared to matched controls of normal tissues and colon adenomas, supporting the notion that SBP1 loss is a late event during tumorigenesis [59]. In addition, changes in the levels of proteins that occur during the progression of human squamous lung cancer were investigated using isobaric tags for relative and absolute quantitation labeling combined with 2D LC-MS/MS [66]. SBP1 levels were determined by western blotting and immunohistochemistry and shown to be progressively lost during bronchial epithelial cancer progression [66].

In contrast to the data obtained examining gastric, bronchial, and colonic tissues, results have also been reported indicating that the reduction in SBP1 expression may be an early event in the evolution of some tumor types, including ovarian cancer [50] and uterine leiomyoma [67]. Huang et al. identified SBP1 to be the most significantly reduced protein in ovarian cancer cell lines, including DOV13, OVCA429, OVCA882, TOV112D, and SKOV3 using a membrane proteome profiling analysis [50]. However, relatively low levels of SBP1 were also observed in the immortalized human ovarian cell line, HOSE2089, indicating that the reduction of SBP1 may have occurred during the immortalization process [50]. SBP1 expression was also approximately 4-fold lower in leiomyoma samples compared to normal myometrium, as determined by western blotting and immunohistochemistry [67]. However, SBP1 levels were similar in tissues obtained from patients with proliferative secretory and atrophic endometrium in either leiomyoma or normal myometrium. These contrasting results may indicate distinct roles of SBP1 in the development of cancers of different origins.

## 5. Physiological Roles for SBP1

The impact of SBP1 on normal biological processes and pathologies other than cancer may be due to roles in the modulation of cellular redox homeostasis. The SBP1 amino acid sequence contains two bis (cysteinyl) sequence motifs, Cys-X-X-Cys, at Cys5-X-X-Cys8 and Cys80-X-X-Cys83 shown to be a characteristic feature among several proteins which are involved in modulating the cellular redox state in vivo [68]. In addition, SBP1 may also modulate the redox state of the extracellular environment. Experimental data in support of this comes from a study where the knockdown of *SBP1* in MCF-7 breast cancer and HC116 colon cancer cells by siRNA resulted in increased levels of H_2_O_2_ and superoxide ion, leading to enhanced apoptosis when cells were exposed to selenite [69]. The authors attributed this effect to the significant increase in extracellular glutathione in the culture media. Changes in either the intracellular or extracellular environment can potentially impact a broad range of biological processes responsive to reactive oxygen in signaling pathways and contribute to the pathology associated with SBP1 dysregulation.

**Figure 2:**
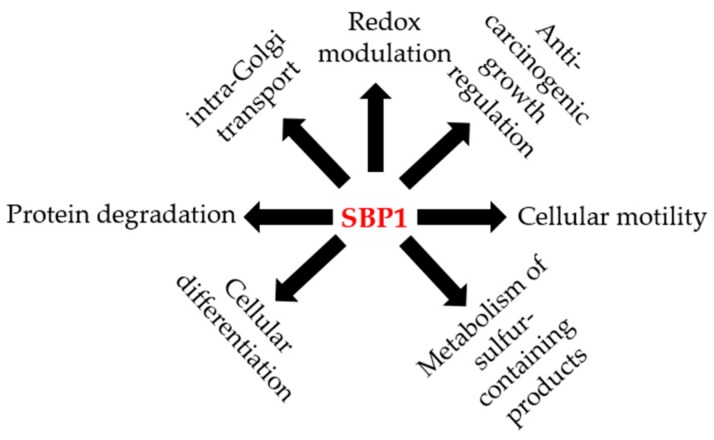
The potential roles of SBP1 in human health and disease. Illustration of the different potential functions reported for SBP1 in the published literature.

SBP1 has also been implicated in the late stages of intra-Golgi transport. Using an in vitro intra-Golgi cell-free transport assay, both endogenous and recombinant SBP1 (rSBP1) exhibited transport activity in the cell-free assay and the addition of antibodies directed against SBP1 abolished this activity [70]. This data indicates that SBP1 may be regulating vesicular intra-Golgi transport, particularly at the docking or fusions stages [70]. The reported roles for SBP1 are summarized in Figure 2.

### Tissue-Specific Roles for SBP1

Several studies have indicated a potential role for SBP1 in neurobiology. SBP1 has been localized at the tips of rapidly extending protrusions in T98G glioblastoma multiforme cells in vitro [71]. Cell protrusive motility, which is tightly associated with actin filament polymerization, is an essential function for multiple cellular processes, including cell proliferation and migration. Monomeric G-actin, but not filamentous F-actin, was shown to be recruited to the SBP1-positive tip, indicating that the recruitment of SBP1 and G-actin at the cell margin precedes actin polymerization [71]. In addition, SBP1 recruitment to the cell margin was observed to precede that of G-actin. The extension of the protrusion will stop when G-actin polymerizes to F-actin at the protruding edges, hence, SBP1 and G-actin disappear from these margins. SBP1 also localized to the growing tips of neurites in SH-SY5Y neuroblastoma cells in vitro [71], possibly indicating a role for SBP1 in neuronal cell outgrowth.

Changes in the levels of SBP1 in neuronal tissues may implicate the protein in several neuropathologies. *SBP1* mRNA has reported to be elevated in the frontal cortex of patients with schizophrenia, indicating a potential specialized role in the pathophysiology of schizophrenia and the central nervous system [72,73,74]. Genetic data has also implicated SBP1 in the risk of schizophrenia as two single nucleotide polymorphisms in the *SBP1* gene (rs2800953 and rs10788804) have been identified as susceptibility loci for schizophrenia in a family-wide association study [75]. This, and a report of plasma SBP1 protein levels being decreased in patients with recent-onset schizophrenia [76], collectively indicate a potential specialized role for SBP1 in the pathophysiology of this disease. Whether these data indicate a role for SBP1 in the proper functioning of the central nervous system or the potential neuroprotective effect of Se against oxidative and excitatory brain damage [77] remains to be determined.

SBP1 may also be involved with the pathogenesis of glaucoma. Elevated levels of SBP1 have been associated with elevated ocular pressure [78]. It was also identified as a differentially expressed gene in datasets comparing transcripts in glaucoma to normal control tissues, which has been verified in a rat model of acute elevated intraocular pressure [79]. SBP1 was also identified as a novel target antigen in patients with Behçet’s disease (BD) with uveitis, where an autoimmune response to retinal antigens is considered to be involved in the pathogenesis of the uveitis in those patients [80,81]. What if any role SBP1 plays in these diseases has not yet been investigated.

## 6. The Transcriptional Regulation of SBP1

A greater understanding of the biological roles of SBP1 could be gained by examining how its expression is regulated. A subtractive hybridization approach was used to identify transcripts that were more abundant in the relatively fast growing PC-3 human prostate cancer cells compared to slow growing LNCaP cells [30]. The low levels of *SBP1* mRNA in LNCaP cells was shown to be due to the down regulation of *SBP1* transcription as treatment with of androgen-sensitive LNCaP cells with dihydrotestosterone (DHT, active form of androgen) reduced the levels of *SBP1* mRNA in a reversible, concentration-dependent manner [30]. A more complicated picture was revealed by the analysis of the effect of androgen on normal ovarian epithelial cells obtained from the scraping of the ovary surface of patients with benign disease, an immortalized cell line, and ovarian cancer cell lines [50]. Treatment of the primary and immortalized cells with DHT reduced the levels of *SBP1* mRNA while SBP1 levels were increased in four tumor-derived cell lines by DHT treatment. The mechanism accounting for the differential response of these cell lines to DHT has not been resolved.

In addition to androgens, *SBP1* expression is also downregulated by estrogen treatment (17-β estradiol) in estrogen receptor (ER)-positive breast cancer cells, but not in ER-negative cells [52]. The suppression of SBP1 expression by transforming growth factor beta (TGF-β) was also observed using a rhesus monkey renal allograft model to identify molecules involved in the pathogenesis of chronic allograft nephropathy (CAN) [82]. SBP1 was absent or markedly reduced in vascular smooth muscle cells in monkey kidney allografts with CAN. Testing growth factors previously associated with graft rejection, including IFNγ, TNFα, and PDGF, only TGF-β blocked the expression of SBP1 in the normal human vascular smooth muscle cell line, CRL-1999 [82]. It is unlikely that the effects of androgens or estrogens on *SBP1* transcription is a direct consequence of the binding of the corresponding receptor to the *SBP1* promoter as there does not appear to be a consensus binding sequence for the receptor/transcription factor.

The mouse *Sbp1* gene has been identified as a direct target gene of the hypoxia-inducible factor-1α (HIF-1α) transcription factor in primary keratinocyte cell cultures [83]. Scortegagna et al. examined HIF-1α gain of function during multistage murine skin chemical carcinogenesis in K14-HIF-1α^Pro402A564G^ transgenic mice. They concluded that HIF-1α was functioning as a tumor suppressor, most likely by upregulating target genes, including *Sbp1*. Four hypoxia response elements were located within 1400 bp of the transcription start site of the human promoter region of *SBP1*, although the demonstration that these were bona fide response elements was not provided [83]. HIF-1α is a central mediator of the cellular response to environmental stresses, such as hypoxia [84]. It is overexpressed in many types of human cancer [85,86,87] and its overexpression is associated with treatment failure and increased mortality in some cancers including cancers of the cervix [88,89], breast [90,91], ovary [92], uterus [93], stomach [94], and brain [95]. It is also associated with decreased mortality in other cancers, including those of the head and neck [96] and non-small-cell lung cancer [97]. The consequences of the changes of HIF-1α levels are cancer-type specific and the accompanying molecular alterations, such as SBP1 reduction/loss, can affect the balance between pro- and anti-apoptotic factors. A study by Huang et al. demonstrated that the decreased expression of SBP1 could lead to a higher GPX1 activity and reduced HIF-1α expression in hepatocellular carcinoma, indicating that SBP1 might exert its tumor suppressive function as a regulator of the tumor redox microenvironment [41].

**Figure 3:**
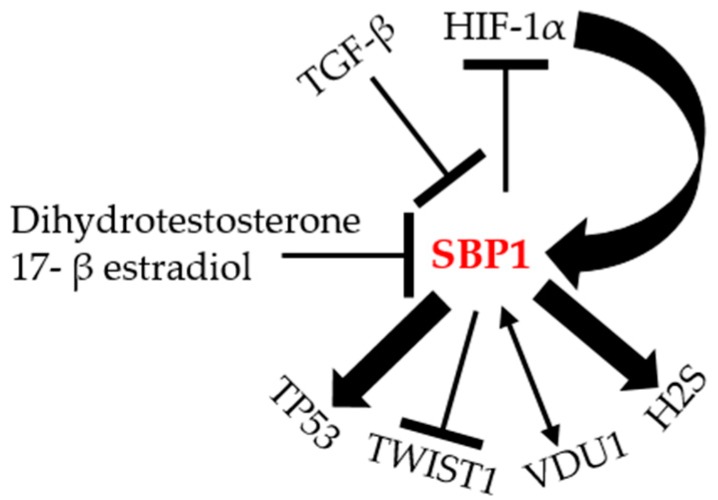
The molecular regulation of SBP1. Illustration of the different proteins that can potentially interact, be regulated by, or regulate SBP1 according to the literature. TGF-β: transforming growth factor beta; HIF-1α: hypoxia-inducible factor-1α; H2S: hydrogen sulfide; VDU1: von Hippel–Lindau protein–interacting deubiquitinating enzyme 1; TWIST1: Twist Family BHLH Transcription Factor 1; TP53: tumor protein p53.

In addition to the putative HIF-1α response elements in the *SBP1* promoter, two potential antioxidant response elements (ARE) with strong homology to the consensus ARE recognition motif are present in promoter region of *SBP1* [28], although the functionality of these sequences as AREs has yet to be established. The presence of functional AREs in the promoter region of *SBP1* may account for the repression of transcription observed when the anti-oxidant selenoprotein GPX1 is ectopically expressed in colon carcinoma cell lines [28], as well as the reciprocal relationship observed in cells and tissues [43]. The factors potentially regulating *SBP1* and some downstream targets are summarized in Figure 3.

In some cases, epigenetic silencing by promoter methylation may be a mechanism by which the expression of *SBP1* is reduced in human colon cancers. Comparing DNA obtained from colon cancer samples to DNA obtained from matched normal tissue indicated significantly more methylation in the promoter region of samples from the cancers [56]. Hypermethylation of the *SBP1* promoter region was demonstrated in the human colon cell lines SW480, Caco-2, HT-29, and HCT1161 in which the extent of promoter methylation was associated with the degree of SBP1 protein levels. Moreover, treatment of these cells with 5-aza-deoxycytidine, a demethylation agent, decreased promoter methylation and resulted in increased promoter activity and protein levels [56]. In contrast, treatment of three different human colon cancer cell lines, LOVO, SNU-C4, and A549, with 5-aza-deoxycytidine did not result in increased SBP1 expression, nor was there any evidence of genetic loss at the *SBP1* locus [59]. There was also a lack of evidence for either hypermethylation or genetic deletion accounting for the low levels of *SBP1* observed in lung cancers [98]. While there is consistent loss of SBP1 in many cancer types, there may be a multitude of ways in which tumor cells can achieve the reduction in SBP1 expression.

## 7. SBP1 Is a Methanethiol Oxidase

The enzymatic function of SBP1 was recently revealed by investigators examining the genetic determinants of extraoral halitosis, bad breath [37]. The authors analyzed breaths and body fluids of five affected individuals with extraoral halitosis from three unrelated families using NMR spectroscopy and gas chromatography with a sulfur-specific detector. All patients exhibited elevated levels of methanethiol (MT), dimethylsulfide (DMS), dimethylsufoxide, and dimethylsulfone in breaths and body fluids [37]. The authors postulated that the accumulation of these compounds was due to a defect in a protein which oxidizes MT, leading to its accumulation in affected individuals. Methanethiol oxidases (MTOs) have not previously been reported in humans, but *SBP1* was identified as a candidate gene for extraoral halitosis by searching for human sequences that were similar to the gene encoding an MT-metabolizing protein previously recognized in methylotropic bacteria, the *mtoX* gene. This effort revealed a 26% identity and a 54% sequence similarity between the two genes [37]. Subsequent sequencing of *SBP1* in patients DNAs revealed four different biallelic mutations in the five patients (1039G>T, 481+1G>A, 673G>T, and 985C>T) that were predicted to be pathogenic. Fibroblasts from these patients had significantly reduced SBP1 protein levels and undetectable MTO enzymatic activity, compared to the control cells [37].

MTO converts MT to H_2_O_2_, formaldehyde, and hydrogen sulfide (H_2_S), the latter is a gaseous signaling molecule with distinct functions at different cellular concentrations [99,100]. At low concentrations, H_2_S stimulates mitochondrial electron transport in mammalian cells, increasing oxygen consumption [101]. At high concentrations, H_2_S is toxic through the inhibition of mitochondrial respiratory-chain complex IV, and consequently reduces oxygen consumption [101]. H_2_S has been proposed as a therapy for multiple disorders by suppressing inflammation, affecting apoptotic pathways, increasing anti-oxidant defenses, and vasodilatation [99,102,103]. It is quite conceivable that many of the consequences of SBP1 expression can be due to the effects on H_2_S levels as well as the other products of the MTO-mediated reaction, on a broad spectrum of physiological endpoints.

## 8. Conclusions

Among the selenium-associated proteins, SBP1 is relatively less studied, but is a highly-conserved protein that may be critical for a variety of physiological functions, including cell differentiation, protein degradation, intra-Golgi vesicular transport, cell motility, and redox modulation. The variety of processes where SBP1 has been implicated to have a role is suggestive that there may be cell type-specific functions that are yet to be identified. The only enzymatic function of SBP1 identified to date is MTO activity and it is possible that different levels of both its substrates and products provide differential signals, resulting in distinct intracellular and extracellular environments for the SBP1-expressing cells. With a better understanding of the function of SBP1 in these tissues, its role in diseases such as cancer may be resolved and SBP1 may become a novel therapeutic target for interventions to control it levels and/or activity.
